# Effects of environmental factors on microbiota of fruits and soil of *Coffea arabica* in Brazil

**DOI:** 10.1038/s41598-020-71309-y

**Published:** 2020-09-07

**Authors:** Tomás Gomes Reis Veloso, Marliane de Cássia Soares da Silva, Wilton Soares Cardoso, Rogério Carvalho Guarçoni, Maria Catarina Megumi Kasuya, Lucas Louzada Pereira

**Affiliations:** 1 Departamento de Microbiologia, Universidade Federal de Viçosa, Avenida P.H. Rolfs S/N Viçosa, Minas Gerais, MG 36570-000 Brazil; 2 Coffee Analysis and Research Laboratory-LAPC, Instituto Federal Do Espírito Santo, Venda Nova Do Imigrante, Rua Elizabeth Minete Perim, S/N, Bairro São Rafael, Espírito Santo, ES 29375-000 Brazil; 3 Instituto Capixaba de Pesquisa, Assistência Técnica E Extensão Rural (Incaper), Av. Domingos Perim, 1231, Providência, Vitória, Brazil

**Keywords:** Environmental microbiology, Biodiversity, Microbial ecology

## Abstract

In recent years, several studies have been developed to understand the impact of fermentation on the final quality of coffee and have indicated that postharvest processing could be a determinant of quality. However, a trend has appeared as a scientific counterpoint, indicating that the interactions between soil, fruit, altitude, and slope exposures with respect to the Sun are important to understand the behavior of the microbiome in coffee. Studies on the microbiota of coffee have addressed its role during the fermentation process, however the knowledge of indigenous microorganisms harbored in fruits and soil of coffee trees growing in fields are essential, as they can contribute to fermentation. Therefore, the aim of this work was to evaluate the influence of topographic and edaphic factors on the bacterial and fungal communities present in the soil and in the fruits of *Coffea arabica* trees. Samples of fruits and soil were collected from different growing areas at different altitudes and soil conditions. The microbial DNA was extracted and sequenced. The results showed the contribution of environmental factors in the structure of bacterial and fungal communities. The richness, evenness and diversity of the mycobiome and bacteriome were higher in the soil than in the fruits, independent of altitude. In addition, coffee trees at higher altitudes tended to have more bacteria shared between the soil and fruits. The co-occurrence/co-exclusion network showed that bacteria-bacteria connections were greater in higher altitudes. On another hand, fungi-fungi and fungi-bacteria connections were higher in low altitudes. This was the first study that evaluates in deep the influence of environmental factors in the microbiota habiting fruits and soil coffee trees, which may affect the coffee beverage quality.

## Introduction

Coffee is one of the most important agricultural commodities in the world. Brazil is the largest exporter and second largest consumer of coffee, producing more than 56 million coffee bags, approximately one third of all coffee exported in the world^1^. The two main coffee species cultivated in Brazil are *Coffea arabica* (75%) and *Coffea canephora* (25%)^1^. While *C*. *canephora* is cultivated in altitudes ranging from 50 to 550 m, *C*. *arabica* crops are present in altitudes from 600 to 1,200 m.

The quality of coffee trees as well as their beverages rely on a suitable combination of climatic and edaphic factors^2–5^. Some of these factors are well known to influence the quality of the beverage; for instance, it is well known that higher elevations produce dense beans with higher quality^5^. If the environment affects the final quality, both processing and environment may be influencing the microbial community structures and hence the chemical composition of the final coffee beans. The studies of coffee ecosystems contribute to a better understanding of a state-of-the-art framework for the further analysis and subsequent control of this complex biotechnological process^6^ since coffee pulp and mucilage are natural substrates for the growth of microorganisms, such as bacteria and fungi, which have been shown to be implicated in coffee quality^7^.

Studies have tried to describe the dynamics of microbiota in coffee crops and processing^8–10^, however, to the best of our knowledge, no published research has investigated in depth the influence of topographic factors such as altitude and sun face exposition on the microbiota of coffee. The microbiota associated with coffee plants may play a critical role in the final quality of coffee, however, the microbial diversity in coffee cherries is still poorly characterized^11^. In fact, most studies on the microbiota in coffee have addressed its role during the fermentation process^6,12,13^ and not the microbiota related to coffee trees growing in fields; it is known that after harvesting, coffee fruits are processed to allow for spontaneous fermentation by indigenous microbiota^13^. Therefore, it is necessary to understand the impact of environmental factors on the indigenous microbiota that inhabits coffee beans, because these microorganisms can develop an important role in the fermentation of coffee beans (e.g. yeasts and lactic acid bacteria)^14^.

Soil microbiota plays an important role in nutrient cycling by making available the required mineral nutrition available for the root system^15^. On the other hand, the microbiota in fruits play an important role during coffee fermentation by degrading the mucilage and impacting the beverage flavor^16^. That the microbial interactions are neither known nor controlled during the fermentation process^17^. Furthermore, there are no studies that compare the shared microbiota between these two niches.

**Figure 1:**
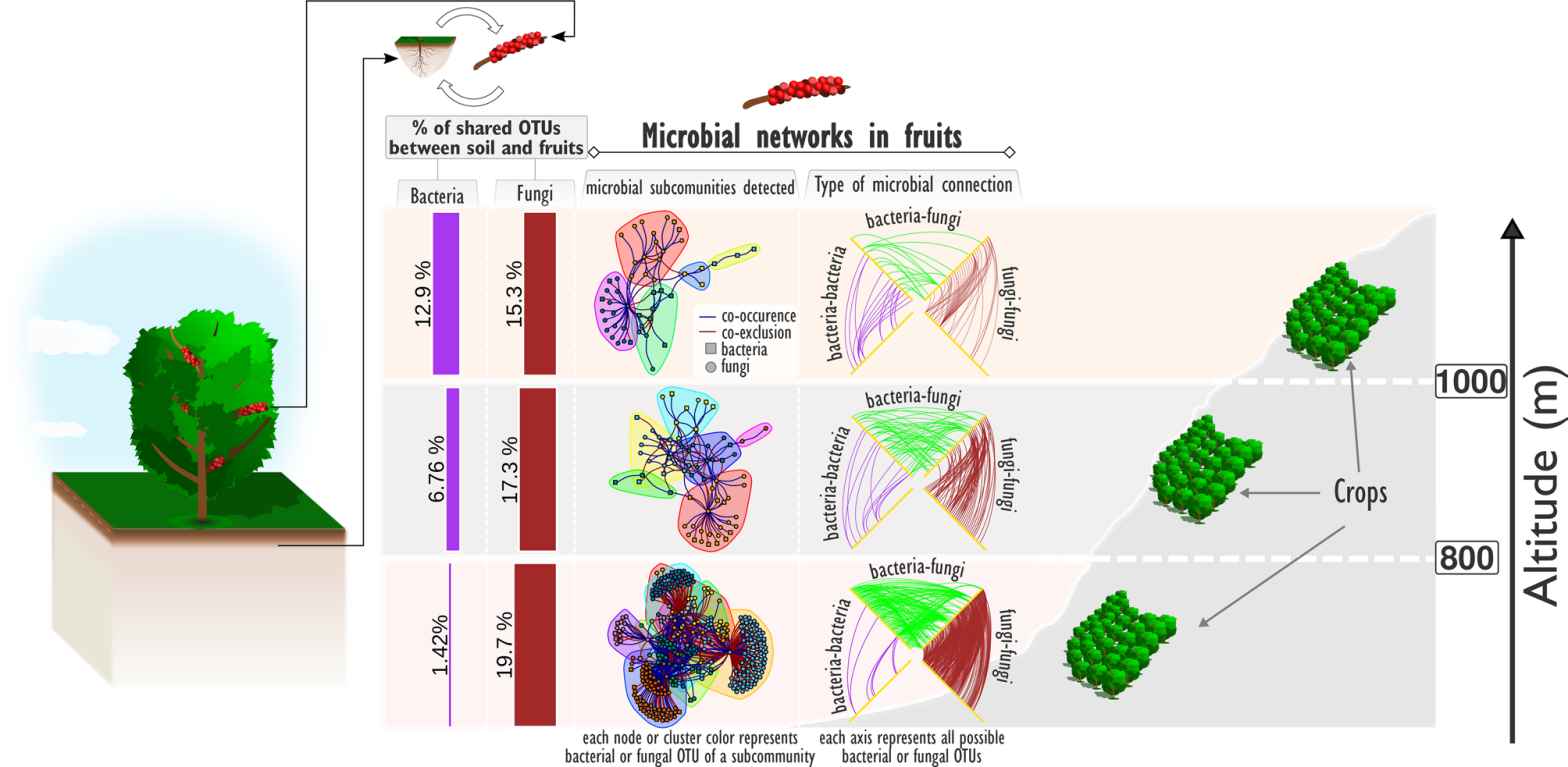
Graphical abstract displaying the main findings of this study.

Considering that soil chemical and topographical factors may influence the microbial composition in coffee crops, the aim of this study was to evaluate the influence of these factors on the bacterial and fungal communities in the soil and fruits of *C*. *arabic*a (see Fig. 1).

## Material and methods

### Study areas and sampling of soil and fruits

As the Espírito Santo is the second largest producer of coffee in Brazil and has many small farms that produce coffee with high quality beverages, in a wide range of environmental factors, it was chose to develop this study. The samplings were conducted on eight small agricultural farms, with altitudes ranging from 735 to 1,078 m (see Supplementary Fig. S1). Red Catuaí 81 was selected because this variety is used by all the farms included in this study.

**Figure 2:**
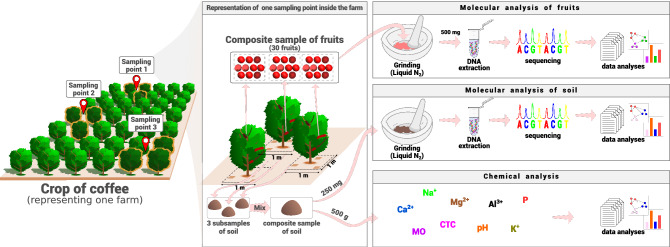
Sampling design of soil and fruits in each crop of coffee. From each farm we collected soil and fruits from three sampling points. The molecular analyses were performed for bacteria and fungi communities.

From each farm, three composite samples of fruits were sampled (Fig. 2); each sample comprised 30 fruits from three coffee plants and three composite samples of soil (about 300 g each), which came from three randomly points inside an area of 1 m^2^ and 10 cm of depth^18^, and under the canopy projection of coffee trees (Fig. 2). All the samples were stored in sterile plastic bags and carried to the laboratory under refrigeration and kept at − 20 °C.

A total of 250 mg of the composite samples was used to soil analysis (Laboratory of Analysis of Soil in Viçosa, MG, Brazil). Soil pH was measured in water (ratio soil:water = 1:2.5). The potential acidity (H + Al) was determined with calcium acetate at pH 7.0. The acid solution Mehlich-1 was used as the extractor of P and K. Ca, Mg and Al were extracted with KCl solution (1 M) and quantified by atomic absorption spectrophotometry. The results allowed the determination the sum-of-bases (SB), base saturation (V), aluminum saturation (m), and potential cation-exchange-capacity (CTC)^19^.

### DNA extraction, PCR and sequencing

DNA extraction from the soil and fruits was performed using NucleoSpinSoil (MACHENEREY-NAGEL) extraction kit. For the soil, 250 mg was used for extraction according to the manufacturer’s instructions. The fruits were first smashed using a sterile pistil and 250 mg of the resulting homogenate was used for the extraction process according manufacturer’s instructions.

To evaluate the mycobiome profile, polymerase chain reactions (PCR) were performed using the primer pairs ITS1F (5′ CTTGGTCATTTAGAGGAAGTAA 3′) and ITS2 (5′ GCTGCGTTCTTCATCGATGC 3′) to amplify the region ITS1 (Internal Transcribed Spacer 1) region of the rDNA of the fungal community, while the bacteriome was evaluated by amplification of V4 subregion of the 16S rDNA with the primers 515F (5′ GTGYCAGCMGCCGCGGTAA 3′) and 806R (5′ GGACTACNVGGGTWTCTAAT 3′)^20^. The PCR libraries were quantified using Qubit hs-DS-DNA kit (Invitrogen, Carlsbad CA) on a Tecan Infinite F200 Pro plate. The results were used to normalize all the libraries in a equimolar concentration of 2 nM, which were pooled for sequencing. The sequencing of the 16S and ITS1 libraries were performed using the platforms Illumina MiSeq 2 × 150 bp and Illumina MiSeq 2 × 250 bp, respectively.

### Processing of 16S amplicons

The reads from sequencing were processed according the pipeline proposed by other authors^21^ using the softwares USEARCH v.11^22^ and QIIME 1.9.1^23^. The reads with an expected error greater than 0.5 were removed using the command *fastq_filter* of the USEARCH v.11. This software was also used to remove the singletons by the command *sortbysize* (minsize = 2)^24^, to remove chimeras by the command *uchime2_ref* and to cluster the remaining sequences in Operational Taxonomic Units (OTUs) using the command *cluster_otus* with a threshold of 97% of similarity. Each OTU was annotated against the SILVA database (Release 132)^25^ using the script *assign_taxonomy.py* available in QIIME 1.9.1. Additionally, a reference tree was reconstructed by the script *make_phylogeny.py*. This tree was used to calculate the distance matrix based in the Unifrac distance^26^.

### Processing of ITS1 amplicons

For the ITS1 dataset, only de forward reads were used since this region is variable in length. The softwares USEARCH v.11^27^, QIIME 1.9.1^23^ and ITSx^28^ were used to process the reads. As performed for 16S sequences, USEARCH v.11 was used to remove low quality sequences and singletons, using the commands *fastq_filter* (-fastq_maxee = 0.5) and *sortbysize* (minsize = 2), respectively. Before clustering the sequences in OTUs, the fungal ITS1 region was extracted. This step ensured the removal of conserved flanking regions 18S and 5.8 s and the removal of ITS sequences from other eukaryotes that are not fungi. The remaining sequences were clustered in OTUs at 97% of similarity with the command *cluster_otus* of USEARCH v.11. The taxonomic assignment was performed using the script *assign_taxonomy.py* of QIIME 1.9.1 with the UNITE (Version 7.2)^29^ database as reference. Due to the high variability of the ITS1 region, which makes the multiple alignment of highly divergent sequences difficult, it was not built a phylogenetic tree, as it was done for 16S.

### Statistical analysis

The Principal coordinate analysis (PCoA) built for the 16S and ITS datasets were based on Bray–Curtis dissimilarity and weighted UNIFRAC indexes, respectively. The topographic and edaphic factors were fitted to the ordination by the *envfit* function available on vegan package 2.5.6^30^. The significance of each edaphic and topographical factor was assessed by PERMANOVA using the function *adonis* in R v.3.5.1^31^. To evaluate the contribution of each group of factors, i.e. edaphic and topographic factors, a RDA variance partitioning^32^ was performed using the function *varpart* of vegan package 2.5.6.

The estimator of richness (Chao1 index), evenness (Pielou’s evenness) and diversity (Simpson index) were calculated using the vegan package v. 2.5.6^30^. To evaluate how these indexes were related with altitude of the crops we fitted linear regressions models using the built-in function *lm* of R v.3.5.1. On another hand, to compare the difference of the values of these indexes among the slope exposures we used the Kruskal–Wallis test using the function *kruskalmc* of package pgirmess v1.6.9 in R v.3.5.1. The putative functions were predicted using FAPROTAX v.1.1^33^.

SparCC correlations^34^ were used to estimate the cooccurrence (positive) and co-exclusion (negative) relationships between bacteria and fungi in different environmental conditions using python scripts with 100 bootstraps using the software fastspar^35^. The cooccurrence networks were built using only significant correlations (p-value ≤ 0.01 and absolute correlation ≥ 0.7). The topology of each network was evaluated using the igraph package^36^.

## Results

### Influence of edaphic and topographical factors on the microbial communities inhabiting fruits and soil of coffee trees

A total of 1,342,396 and 3,135,343 sequences were obtained from the 16S (Archaea and Bacteria) and ITS1 (Fungi) regions, respectively. After cleaning the data, 666,585,16S reads and 1,017,668 ITS1 reads were retained. The raw reads were submitted to the the NCBI-SRA archive and are available in the BioProject PRJNA626678.

**Figure 3:**
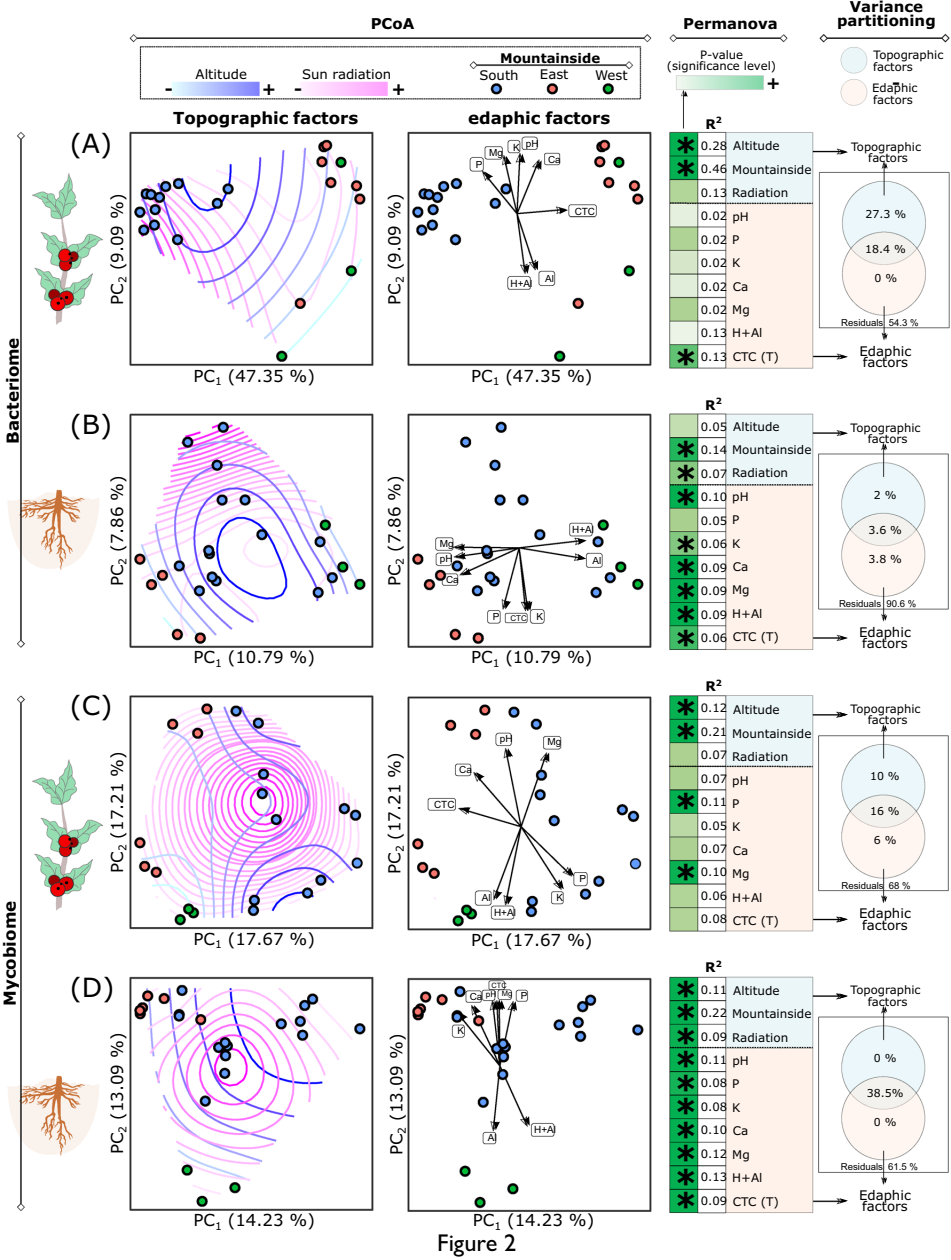
Effect of topographic and edaphic factors on bacteriome and mycobiome of fruits and soil of coffee trees. Analysis of bacteriome (**A**,**B**) and mycobiome (**C**,**D**) in the fruits (**A**,**C**) and soil (**B**,**D**) in the *Coffea arabica* crop. Ordination analysis using Principal coordinate analysis (PCoA) based on Bray–Curtis dissimilarity of OTU abundances. The *envfit* function was used to calculate the fitted curves of topographic and edaphic variables. The significance of edaphic and topographical factors were calculated by PERMANOVA, and the variables assigned by *.

The principal coordinate analysis (PCoA) and a permutational multivariate analysis of variance (PERMANOVA) showed a significant influence of the slope side (p-value = 0.001) and altitude (p-value = 0.001) in bacteria inhabiting fruits (Fig. 3A). Solar radiation influenced the bacterial community in the soil (Fig. 3A) but not in the fruits (Fig. 3B).

The redundancy analysis (RDA) variance partition showed the relative contribution of environmental factors in the structure of bacterial and fungal communities (Fig. 3). In the fruits, the topographical factors influenced the bacterial (Fig. 3A) and fungal (Fig. 3C) communities more than the edaphic factors. On the other hand, in soil, the bacterial community was more influenced by the edaphic factors than the topographical factors (Fig. 3B), whereas the fungal community was equally influenced by both (Fig. 3D). However, in all the cases a high percent of the variance was not explained by these two groups of factors (residuals). It indicates that other factors, which were not considered here, could also be shaping the microbial communities, mainly the bacterial community of soil (residuals = 90.6%).

**Figure 4:**
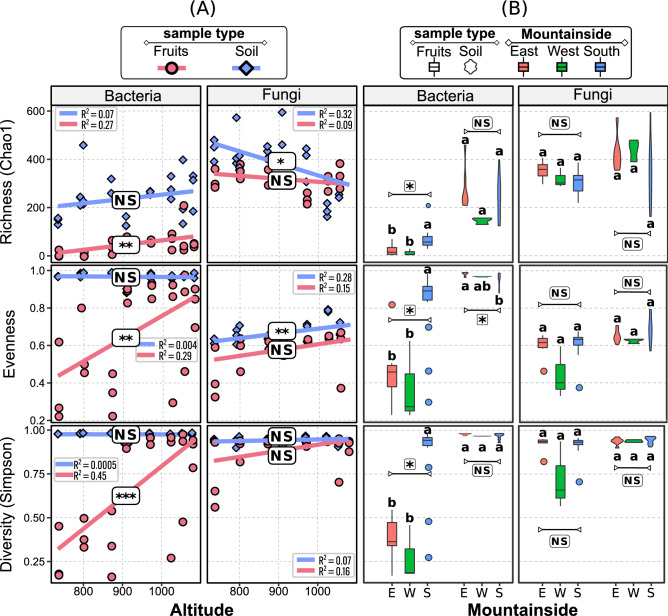
Estimated richness (Chao^1^), evenness and diversity (Simpson index) of the bacteriome and mycobiome in the fruits and soil of *Coffea arabica*, growth at (**A**) different altitudes and (**B**) sun-facing slopes. For altitude, NS, *, ** and *** stand for nonsignificant, significant by t-test at 0.05, 0.01 and 0.001, respectively, and for sun-face, NS, *, ** and *** indicate nonsignificant, significant at 0.05, 0.01 and 0.001, respectively, by Kruskal–Wallis test. E = east; W = west; S = south.

To evaluate the differences in richness, evenness and diversity among different altitudes and slope exposures with respect to the Sun, the Chao1 estimate of richness, the Pilou evenness and the Simpson diversity index were calculated for each sample. These three indexes of the bacteriome and mycobiome were higher in the soil than in the fruits in most altitudes (Fig. 4A). The bacterial diversity in the fruits increased with the altitude (p-value < 0.001), which was explained by the increase of evenness (p-value < 0.01) and richness (p-value < 0.01).

In the soil, these bacterial indices were not influenced by altitude (Fig. 4A). On the other hand, the richness and evenness of fungi were influenced in soil but not in fruits (p-value < 0.01; Fig. 4A). These results show that bacterial richness increased at higher altitudes while the fungal richness decreased. The slope exposure with respect to the Sun also influenced these three indexes of the bacteriome of fruits, but not the mycobiome (Fig. 4B). These three indexes were higher for the bacterial communities inhabiting fruits in south-facing slopes (p-value < 0.05) than those in the east and west, except for the richness. In soil, only the evenness of bacterial community present in the south-facing slope were smaller (Fig. 4B).

### Taxonomic composition and OTUs shared between fruits and soil in different altitudinal zones

We delimited three altitudinal zones: low altitudes (< 800 m), median altitudes (between 800 and 1,000 m) and high altitudes (≥ 1,000 m). For each zone, it were evaluated the taxonomic composition and bacteria shared between soil and fruits.

In all altitudinal zones, the most abundant bacterial phylum was Proteobacteria (see Supplementary Figs. S2 and S3). This group was more predominant in soil (ranging from 76 to 98% of total number of reads) than in fruits (ranging from 35 to 98%). The candidatus phyla Latescibacteria and WPS-2 were found exclusively in fruits. Regarding to composition of fungal community, Ascomycota was the most abundant phylum in both soil and fruits. We identified a total of five phyla in fruits (Ascomycota, Basidiomycota, Chytridiomycota, Mortierellomycota and Mucoromycota) and eleven in soil (Ascomycota, Basidiomycota, Blastocladiomycota, Calcarisporiellomycota, Chytridiomycota, Entomophthoromycota, Entorrhizomycota, Glomeromycota, Mortierellomycota, Mucoromycota and Rozellomycota). A large amount of sequences (24 to 36% in fruits and 16 to 22% in soil) could not be assigned to any known fungus from the UNITE database (see Supplementary Fig. S2).

**Figure 5:**
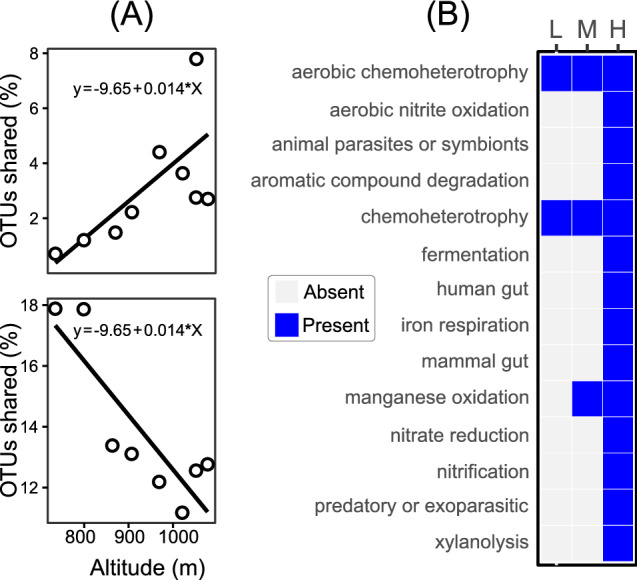
Shared OTUs between fruit and soil. (**A**) Linear regression of shared Operational Taxonomic units (OTUs) of bacteria and fungi, between fruits and soil of *Coffee arabica*, growing at different altitudes. *Linear coefficients followed by “*” indicate statistical significance at 0.05 of probability by Student’s t-test. (**B**) Bacterial functional groups predicted using Functional Annotation of Prokaryotic Taxa (FAPROTAX) in each altitudinal zone. L = Low altitudes (≤ 800 m); M = Median altitudes (> 800 m and ≤ 1,000 m); L = High altitudes (> 1,000 m).

Since the altitude was previously reported as an important factor to coffee’s flavor^37^ and we have found that both bacterial and fungal communities were affected by this variable (Fig. 3), we evaluated the percent of shared fungi and bacteria between soil and fruits among these three zones of altitude. We found that while altitude increased, the percent of shared bacterial OTUs decreased and fungal OTUs increased (Fig. 5). Additionally, we performed a functional profile prediction of these shared bacteria using FAPROTAX (Fig. 5) and found an increase of functional roles in high altitudinal zones. Some shared functions were exclusively found in the highest altitudinal zone (> 1,000 m) like aromatic compound degradation and nitrate reduction (Fig. 5).

### Complexity of co-occurrence networks among different zones of altitude

**Figure 6:**
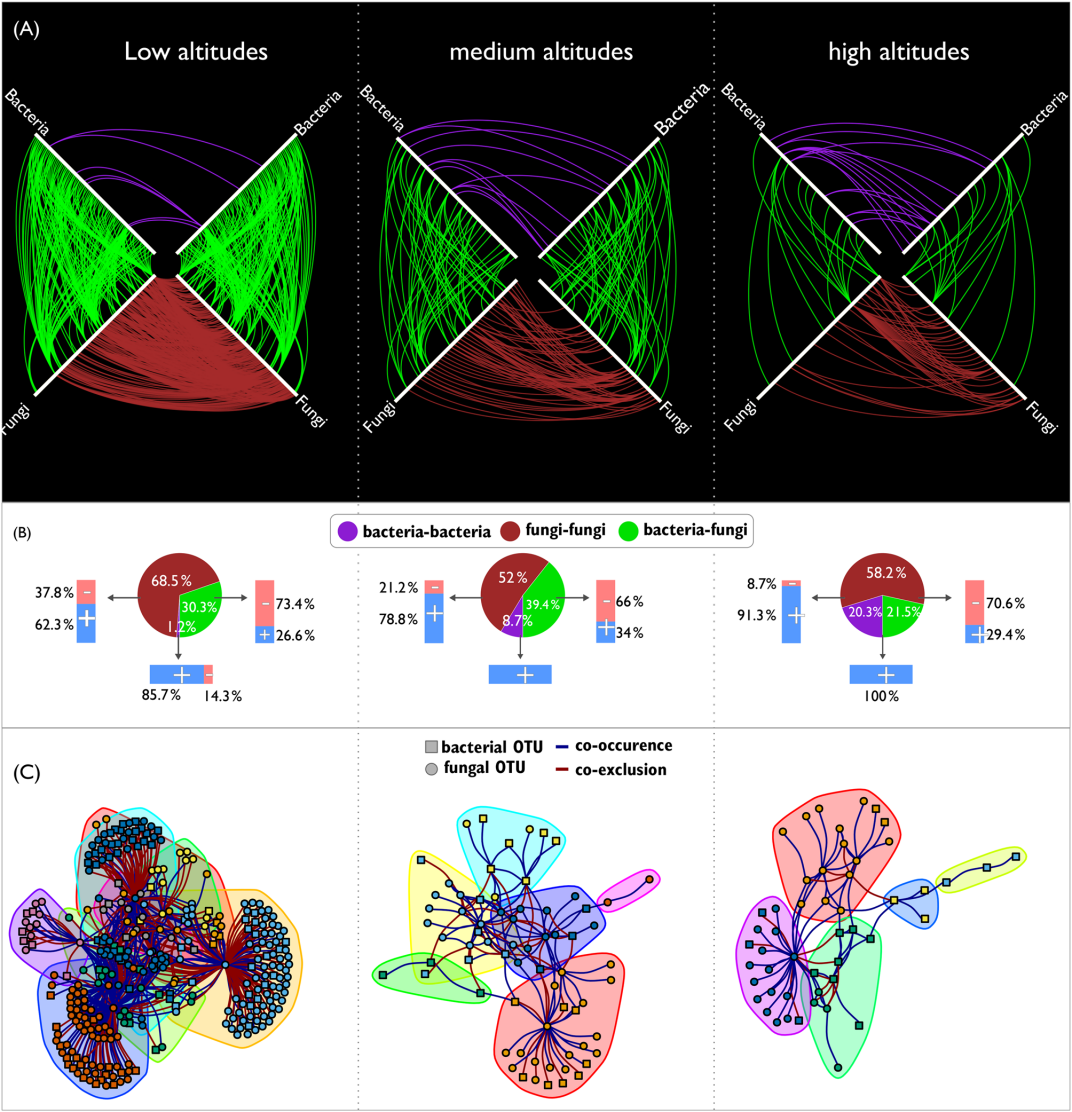
Co-occurrence/co-exclusion network of microbiome in fruits at three altitudinal levels. (**A**) Hive plot of co-occurrence/co-exclusion network of microbiome in fruits at three altitudinal zones: low altitudes (≤ 800 m), median altitudes (> 800 m and ≤ 1,000 m), high altitudes (> 1,000 m) based on significant SparCC correlations (p-value < 0.01 and absolute correlations > 0.7). Bacterial and fungal OTUs were displaced along each one of the white axes. Edges colored with blue, red and green represents, respectively, co-occurrence or co-exclusion of type bacteria-bacteria, fungi-fungi and bacteria-fungi. (**B**) Pie charts shows the percent of each type of interaction (i.e. bacteria-bacteria, fungi-fungi and bacteria-fungi) and bar plots the percentage of positive (co-occurrence) and negative (co-exclusion) connections of each type. (**C**) Microbial communities at each altitudinal zone. Each cluster in C represents a community detected by the function *make_clusters* function in the igraph package. Each node represents a 97% identity operational taxonomy unit (OTU). Blue and red edges represent positive and negative cooccurrence of OTUs linked by them. Each node was labeled at the class level.

In addition to previous analysis we evaluated the co-occurrence and co-exclusion among OTUs in the three altitudinal zones. We built a co-occurrence network using only statistically significant SparCC correlations (p-value ≤ 0.01 and absolute correlation ≥ 0.7)^34^. The number of connections were higher in fruits at low than in high altitudes, however, the number of connections bacteria-bacteria increased in higher altitudes (Table 1 and Fig. 6A). The majority of connections took place among fungi, followed by bacteria-fungi and bacteria-bacteria (Fig. 6B and Table 1). While the majority of connections among OTUs from the same group (i.e. *bacteria-bacteria* or *fungi-fungi*) were positive (i.e. co-occurrences), the majority of connections among OTUs of different groups were negative (i.e. co-exclusion). Using a community detection algorithm (*cluster_walktrap* function of igraph package^36^), we found eight, six and five communities at low, median and high altitudes, respectively (Fig. 6C and Table 1).

**Table 1:** Topological properties of microbial networks in fruits of *Coffea arabica*.

Altitude (m)	Diameter	Number of nodes			Edges							Centrality^a^	Modularity^b^	Number of communities^c^
		All	Bacteria	Fungi	All	Bacteria–bacteria			Fung–fungi					
						No	+ (%)	− (%)	No	+ (%)	− (%)			
≤ 800	5	235	9	226	407	7	85.7	14.3	400	62.3	37.8	0.42	0.49	9
> 800 and ≤ 1,000	4	54	13	41	77	11	100.0	0.0	66	78.8	21.2	0.27	0.50	7
> 1,000	7	45	13	32	62	16	100.0	0.0	46	91.3	8.7	0.41	0.59	5

^a^Calculated with the *centr_degree* function of the *igraph* package.

^b^Calculated with the modularity function of the *igraph* package.

^c^Each community corresponds a group of nodes that are more densely connected among them than the rest of network. Calculated witht the *cluster_walktrap* function of the *igraph* package.

## Discussion

This was the first study to evaluate in depth the influence of environmental factors on the microbiota inhabiting coffee fruits and soil; other studies have described the microbiota in coffee fruits or in soil, without correlating them^8,9,11^. Furthermore, other studies have focused on the microbiota during the fermentation of coffee^6,12,38^, not in the microbiota present in situ.

The differences found in structure (Fig. 3) and diversity (Fig. 4) of microbiota in fruits and soil indicate that climatic and microclimatic alterations can directly influence the microbiota associated with coffee, as reported by other authors^39^. For example, the structure of the bacterial community can vary as a function of slope exposure, since topographic factors cause differences in the microclimate, especially in soil temperature, which correlates with the carbon and nitrogen content of the soil^40^. Additionally, the intensity of the light, the temperature and the electrical conductivity of the soil are influenced by the elevation of the mountain slope, cultivation system and/or an interaction of the two, and these factors affect the soil macrofauna in coffee plantations^41^.

The richness and diversity (Fig. 4) observed here were higher than those in previous studies^9,11^. The condition of the environment, like the incidence of solar radiation, can lead to changes in the internal metabolites, creating a stress conditions and consequently different conditions that may affect the development of microorganisms^2^, and the position and altitude of the fields were the main variables that influence coffee quality^42^. Here we did not find change in richness, evenness or diversity of bacteria in soil along the altitude (Fig. 4). However, we found an increase of bacterial diversity in fruits at high altitudes. The bacterial predominance has been observed in soil of organic coffee systems in India^43^, and the relative bacterial abundance increased at higher altitudes, which was related to increasing levels of soil organic matter and nutrients with altitude^44^. The boost of this diversity at high altitudes might be due to the greater contribution of soil as a source of bacteria than the soil at low altitudes. Soil can act as a source of bacteria for bacteria endophytes^45^ and the functional prediction of these shared bacteria (Fig. 5) reveals that soil in high altitudes furnishes bacteria with functional properties that can contribute to the fermentation of fruits in the postharvest processing. Once coffee beans at high altitudes presents more mucilage/water ratio^46^ and more fat content^47^, the presence of an bacterial community with a high functional diversity can improve the processing of compounds by providing enzymes and compounds that can be useful for the fermentation process of coffee mucilage^46^.

For the first time, the co-occurrence/co-exclusion network of microbiota in coffee fruits was investigated (Fig. 6). Most connections between fungi- fungi and bacteria-fungi were found in low altitudes while bacteria-bacteria connections were greater in high altitudes (Fig. 6). Inoculation of coffee at higher altitudes is not as effective as that performed at lower altitudes^2,48^. Since the fermentation process might be affected by the indigenous microbiota^49^, the microbial networks connections in each altitudinal zone might affect the growth of the inoculum. Thus, the study of microbial ecology of coffee is essential to fully understand the production and conversion of beneficial metabolic precursors that provide high-quality brewed coffees and their unique flavors^11^. This information can be related to the sensorial quality of coffee from those trees growing at high altitudes, with an annual rainfall less than 1,500 mm^47^ and with open or medium shading^50^.

Two OTUs closely related to *Colletotrichum kahawae*, which causes coffee berry disease, were not found in higher altitudes, although this disease be often in this condition^51^. Bacterial networks with high connectance can inhibit the attack of pathogens due to increase of niche overlap between indigenous microbiota and pathogen, which increases the resources competition^52^. The increase of bacteria-bacteria connections in higher altitudes might be due to reduction of the connections fungi-bacteria which the most were negative, so that fungi could be inhibiting bacteria to interact to each other (Fig. 6B).

These results allow us to understand the influence of the environment on both bacteriome and mycobiome. This understanding suggests that the final quality of coffee beverages might be determined by the microbial diversity/interaction, which is influenced by the environmental condition where the crops are being grown, which may affect the coffee beverage quality.

## Supplementary information

Supplementary Information.
