# Supplementary material for: Effects of environmental factors on microbiota of fruits and soil of Coffea arabica in Brazil
Source: Sci Rep. 2020 Sep 7;10:14692. doi: 10.1038/s41598-020-71309-y (PMC7477199; doi:10.1038/s41598-020-71309-y)
Supplement: Supplementary file 1 — Supplementary Information. [file 41598_2020_71309_MOESM1_ESM.pdf]

**Supplementary information**

# Effects of environmental factors on microbiota of fruits and soil of *Coffea arabica* in Brazil

Veloso, Tomás Gomes Reis[1]; da Silva. Marliane de Cássia Soares[1]; Cardoso, Wilton Soares[2]; Guarçoni, Rogério Carvalho[3]; Kasuya, Maria Catarina Megumi[1*], Pereira, Lucas Louzada[2]

**Affiliations:**

1.Universidade Federal de Viçosa, Departamento de Microbiologia, Viçosa, Minas Gerais. (Avenida P.H. Rolfs S/N Viçosa, Minas Gerais - MG, 36570-000).

2. Instituto Federal do Espírito Santo, Coffee Analysis and Research Laboratory - LAPC, Venda Nova do Imigrante, Brazil. (Rua Elizabeth Minete Perim, S/N, Bairro São Rafael, Espírito Santo - ES, 29375-000).

3. Instituto Capixaba de Pesquisa, Assistência Técnica e Extensão Rural (Incaper). Av. Domingos Perim, 1231 – Providência

**[*]Corresponding author**

Maria Catarina Megumi Kasuya

E-mail: catarinakasuya@gmail.com

Phone: (+55) 31 3612- 2456

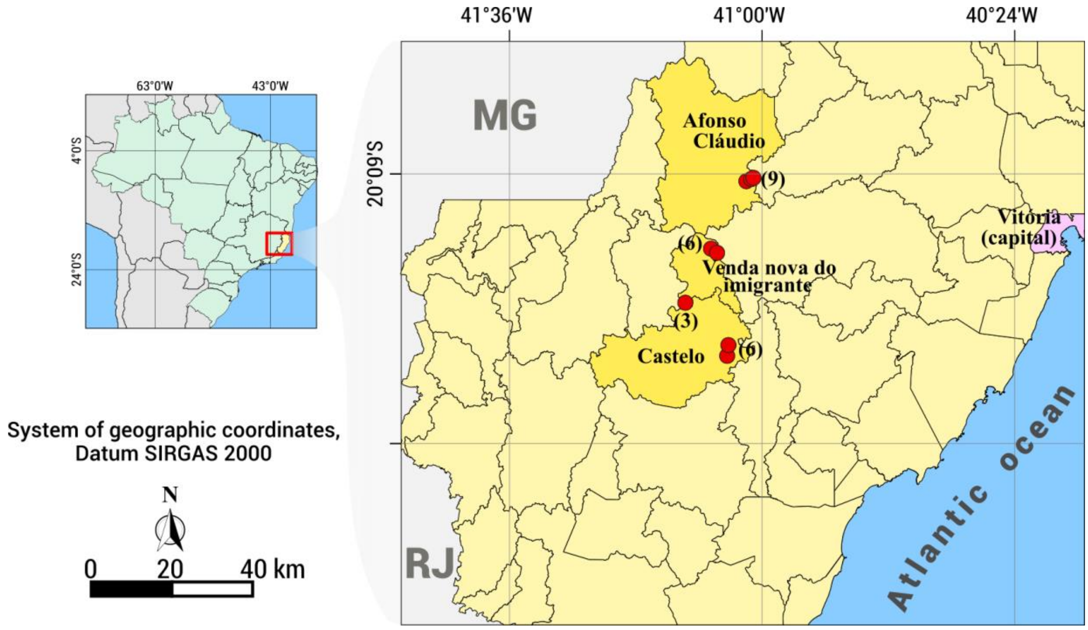


**Supplementary Figure S1**: Sampling sites in the state of Espírito Santo, Brazil. A total of 24 samples were collected. *The number in parentheses show the number of samples in each region because some points overlap. RJ = Rio de Janeiro state. The map was generated by the software QGIS version 3.4.11 (https://qgis.org/en/site).

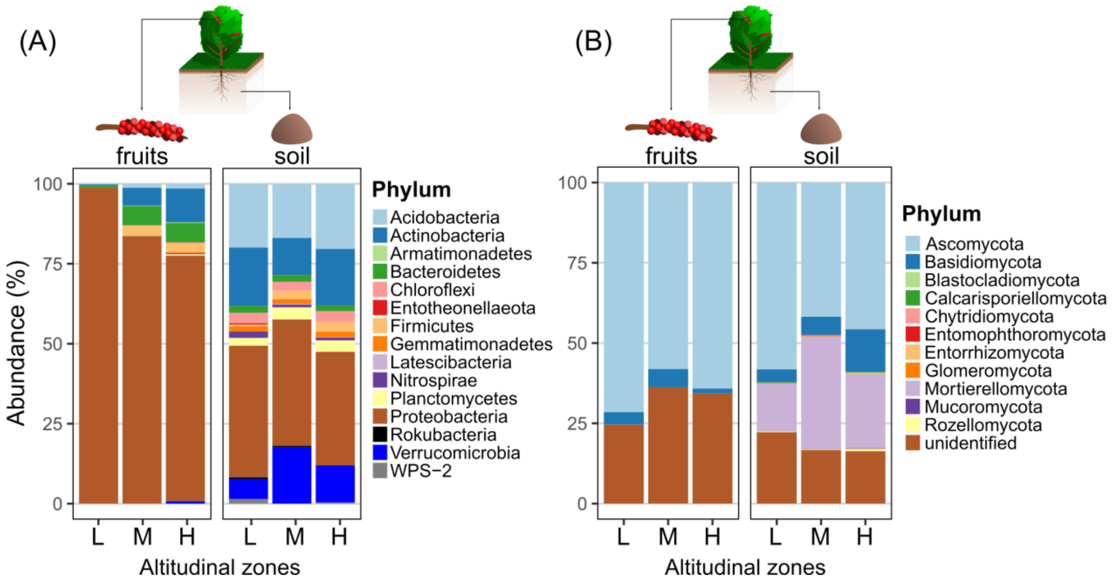


**Supplementary Figure S2**: Percentage of phyla of the (A) bacterial and (B) fungal OTUs found in the fruits and soil of coffee crops along a wide range of altitudes in Espírito Santo, Brazil. L = Low altitudes (≤ 800 m); M = Mediam altitudes (> 800 m and ≤ 1000 m); L = High altitudes (> 1000 m).

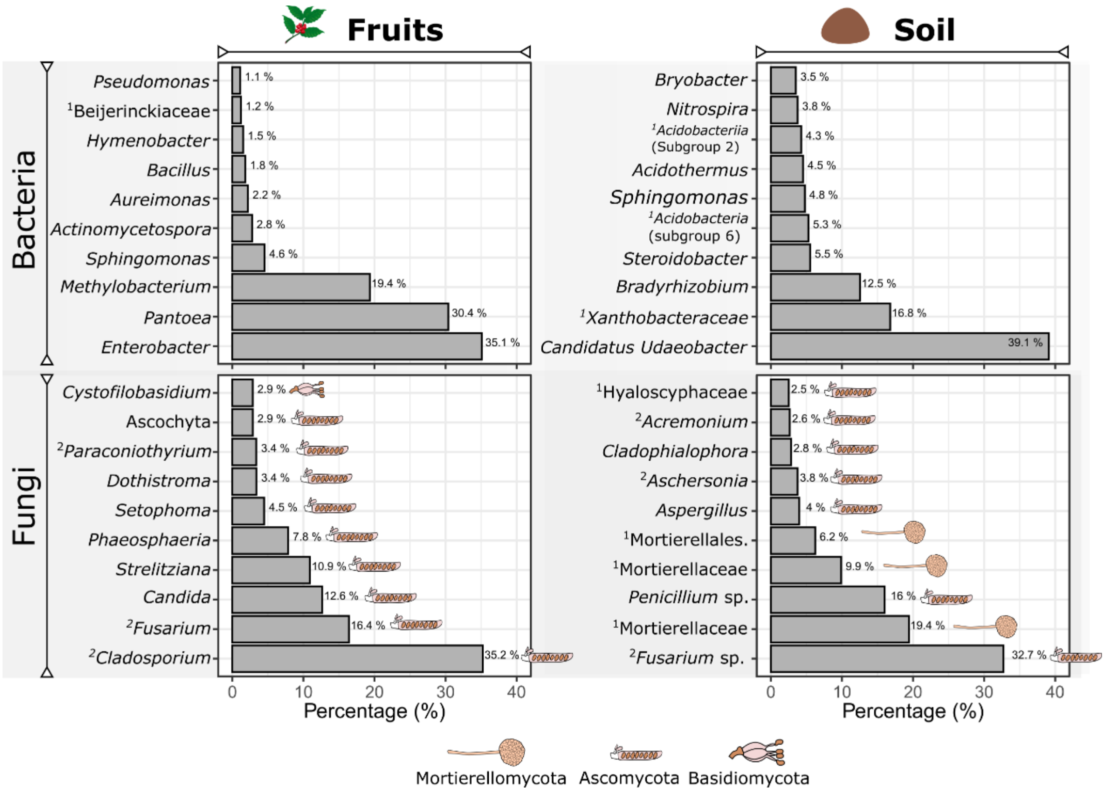


**Supplementary Figure S3:** Ten most frequent OTUs of the bacteria and fungi found in fruits and soil of coffee crops. [1] Identification was not performed at genus level due the absence of a suitable similar sequence in the databases (UNITE and GenBank).[2]Sequences annotated with the GenBank database because the annotation with UNITE did not provide resolution at the genus level.
